# Are stethoscopes risky in COVID-19?

**DOI:** 10.1136/postgradmedj-2020-138085

**Published:** 2020-05-21

**Authors:** Yuk-Fai Lau, William Wei, Chu-Pak Lau

**Affiliations:** Department of Ear, Nose and Throat, Pamela Youde Nethersole Eastern Hospital, Hospital Authority, Kowloon, Hong Kong; Medicine, University of Hong Kong, Hong Kong, Hong Kong; Medicine, University of Hong Kong, Hong Kong, Hong Kong

**Keywords:** cardiology, infection control, audiology

A stethoscope is an extensively used piece of medical equipment for the doctors, nurses, physiotherapists and other allied professionals. Stethoscopes spread of infection between patients and between users,^1  2^ and a patient-specific stethoscope has also been recommended. However, adherence to stethoscope cleaning by the medical professionals is low.^2^

The Littmann classic III stethoscope has a detachable rubber ear tip piece that fits the external ear. To avoid transmitting infection, the bell and diaphragm can be cleaned with alcohol, and the plastic ear tip piece can be detached for thorough cleaning although it is not alcohol resistant. Only one size ear tip piece is available for all stethoscope types despite a smaller diameter metal stem for the classic III. Vigorous alcohol scrubbing and turning can loosen and detach the ear tip pieces.

**Figure 1:**
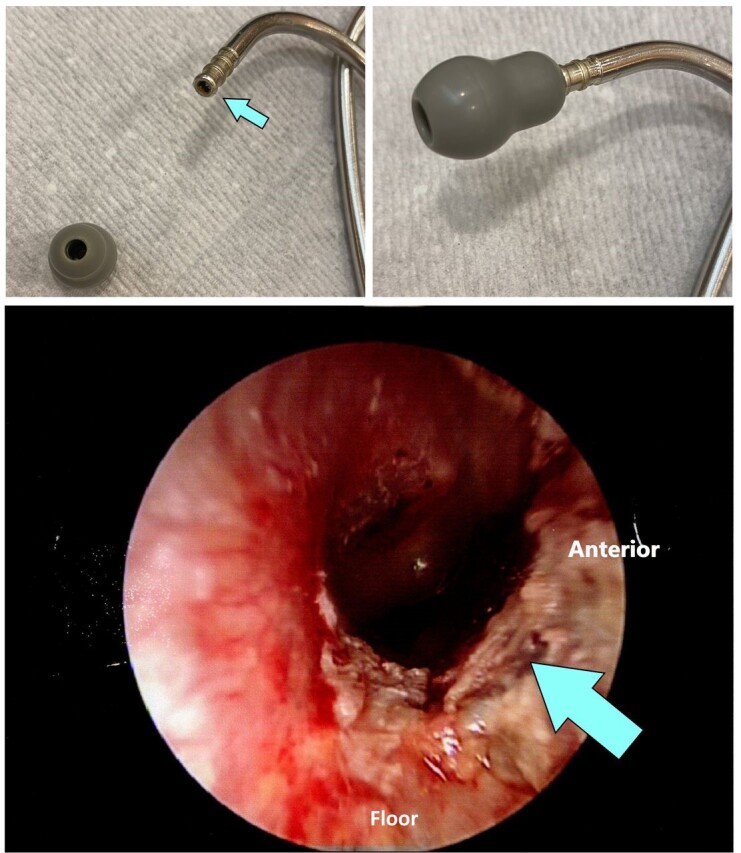
Metal bevel end (small arrow) exposed when soft plastic ear tip dislodged. this caused a semicircular flap in the right external auditory meatus(large arrow).

We report an unusual risk of the stethoscope. A doctor (CPL) suffered from a right external auditory canal (EAC) injury after using such a patient stethoscope that had a loose ear tip. The right ear tip inadvertently dislodged when applied, and the bare metal ear tip acted like a bevel to traumatise his EAC (figure 1). The doctor experienced severe otalgia with blood stained otorrhoea. Examination revealed a semicircular abrasion of the right EAC, but the tympanic membrane was intact. The patient recovered after treatment with Pope ear wick, levofloxacin ear drops and systemic antibiotic. This highlights the importance of checking the integrity of stethoscopes after disinfection. Safe medical practice is still utmost important, in particular during COVID-19 outbreak.

The safety of stethoscope use for respiratory auscultation in COVID-19 pandemic has been questioned.^3^ Lung ultrasound was suggested to replace the stethoscope. Ultrasound probe can be wireless and can be covered with disposable plastic sheet covers. It is more difficult to cover the stethoscope with specific protection sheets without compromising its use.

Cardiac involvement in hospitalised patients with COVID-19 is common, myocardial injury (7%–17%), myocarditis and acute coronary syndrome have been reported. Cardiac arrhythmias may occur de novo or after treatment with QT prolonging drugs.^4^ While a stethoscope can be a useful piece of equipment, alternative technology, such as a handheld ultrasound scanners with disposable protective covers may be a good alternative, and more informative of conditions such as pericardial effusion, and cardiac and valvular function and valvular disease. The role of stethoscope in the era of evolving new technologies has long been contested.^5^
